# Supplementary material for: The AICL-KLRF1 axis supports CD4-CD8 T cell communication and cytokine competence in pre-exhausted CD8+ T cells
Source: EMBO Rep. 2026 Mar 18;27(8):2029–60. doi: 10.1038/s44319-026-00732-5 (PMC13121605; doi:10.1038/s44319-026-00732-5)
Supplement: Supplementary file 1 — Appendix [file 44319_2026_732_MOESM1_ESM.pdf]

Barone *et al.*

The AICL-KLRF1 axis supports CD4-CD8 T cell communication and cytokine competence in pre-exhausted $CD8^+$ T cells

Appendix

Table of Contents:

Appendix Figure S1	Page 2
Appendix Figure S2	Page 4
Appendix Table S1: Cell culture	Page 6
Appendix Table S2: Flow stain KLRF1-receptor inhibition	Page 7
Appendix Table S3: Flow stain AICL upregulation	Page 8
Appendix Table S4 Sorting stain	Page 9
Appendix Table S5 IMC panel	Pages 10-11
Appendix Table S6 IMC Metadata	Page 12
Appendix Table S7 scRNA-seq IFNg primer	Page 13
Appendix Table S8: Gene sets used to calculate Tpex and Tex scores in Figure EV 5	Page 14

Appendix Figure S1: Gating of CD4⁺ AICL⁺ populations (Figure 3C)

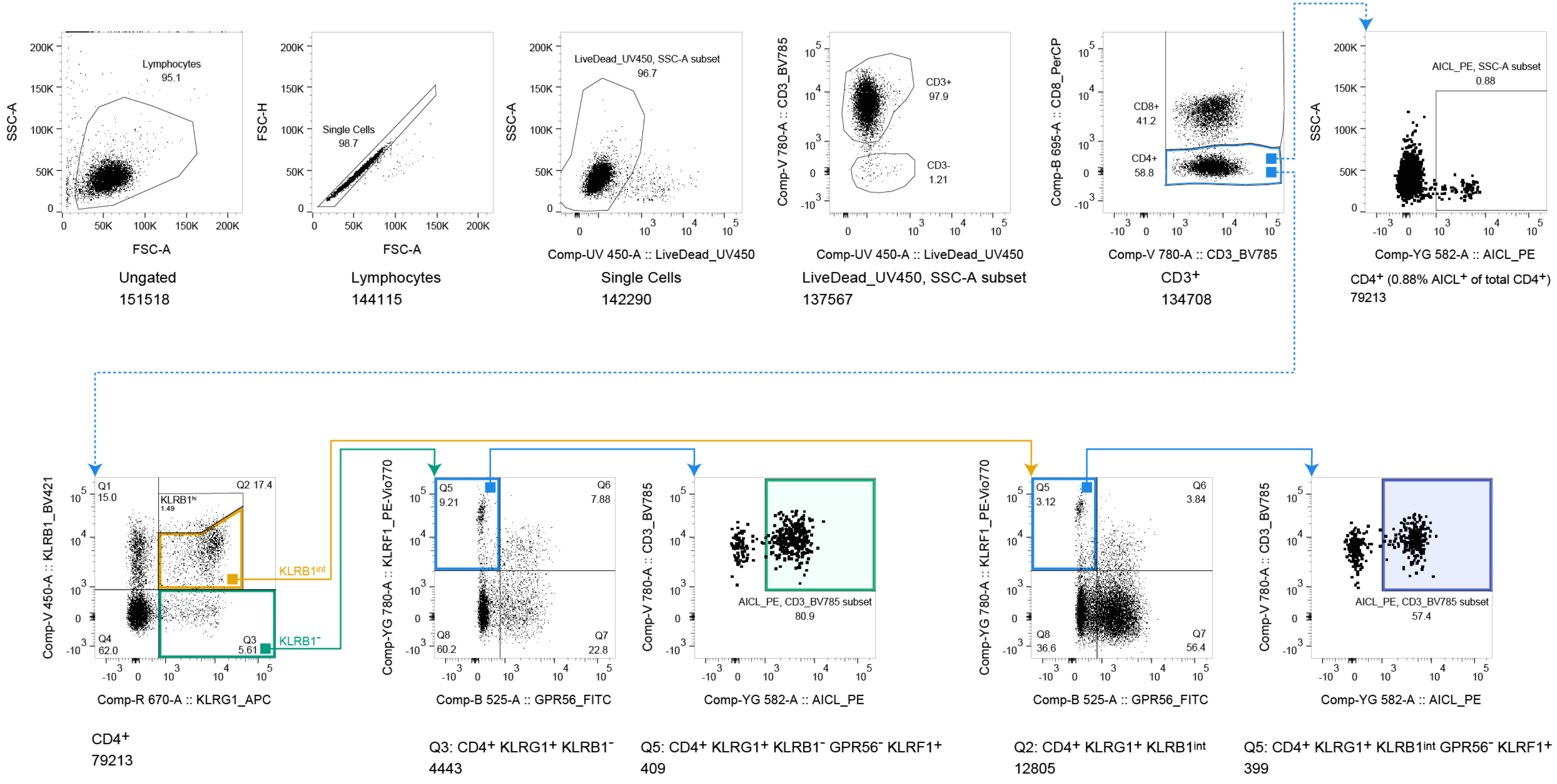

CD4⁺ KLRG1⁺ KLRB1⁻ GPR56⁻ KLRF1⁺

AICL⁺: 331 cells
0.42% of total CD4⁺

CD4⁺: 79213 cells

CD4⁺ KLRG1⁺ KLRB1^{int} GPR56⁻ KLRF1⁺

AICL⁺: 229 cells
0.29% of total CD4⁺

CD4⁺: 79213 cells

Appendix Figure S1: Exemplary gating strategy into two $CD4^+$ (blue, second row) populations $CD4^+$ $KLRG1^+$ $GPR56^-$ $KLRF1^+$ $KLRB1^-$ (green, second row) and $CD4^+$ $KLRG1^+$ $GPR56^-$ $KLRF1^+$ $KLRB1^+$ (yellow, second row) T cell populations. The AICL+ fraction of each subset (third row) was calculated relative to the parent $CD4^+$ T cell gate as indicated in the boxes below the dot plots. Additionally, the total $AICL^+$ population of the CD4 gate is shown in the second row on the right hand side.

Appendix Figure S2: Boolean Gating Strategy of IMC dataset and Annotation of non-B/non-T immune compartment (Figure EV 2)

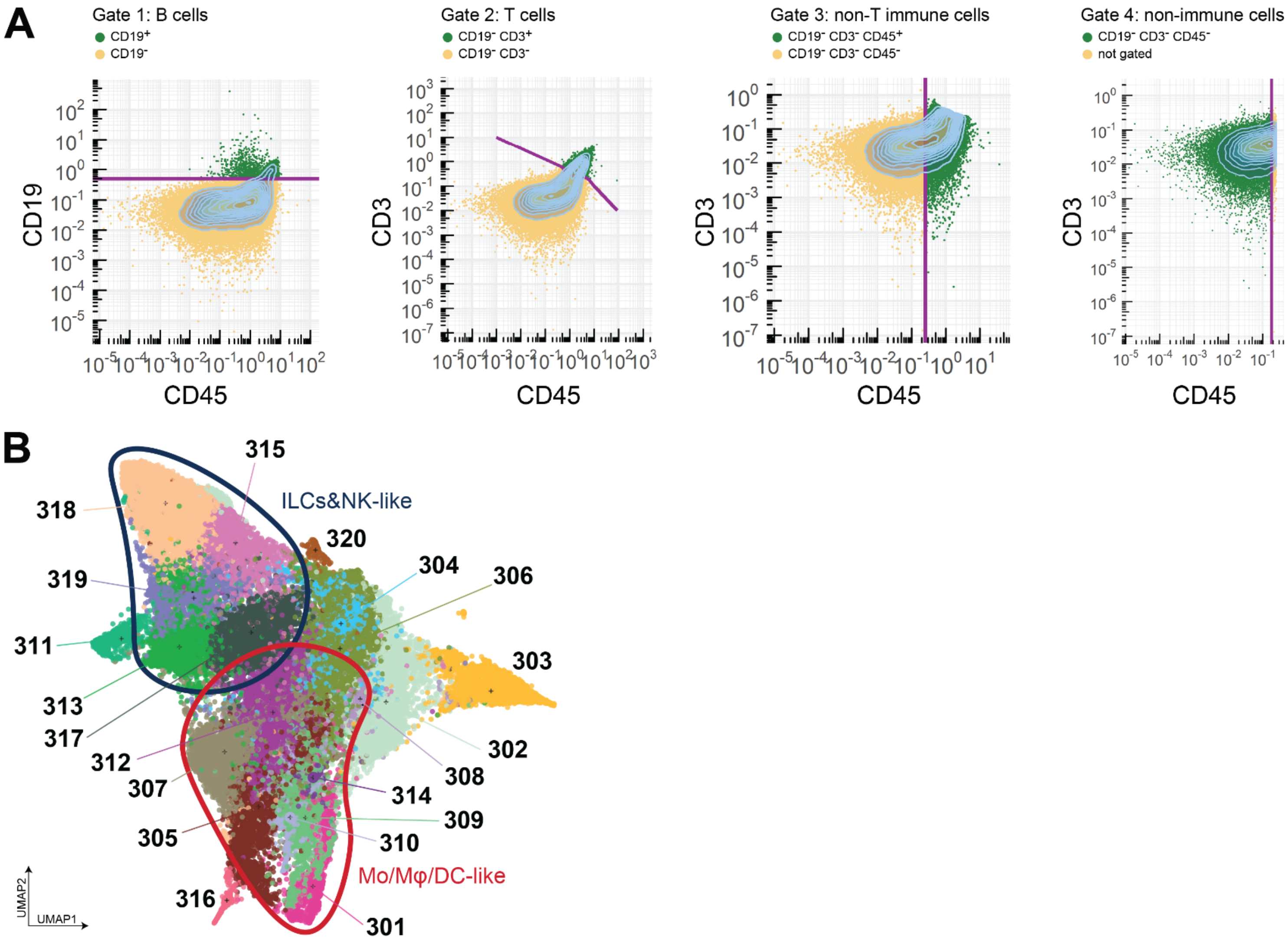

Appendix Figure S2: A) Boolean gating strategy of the IMC dataset to separate $CD19^+$ B cells (Gate 1), $CD19^-$ $CD3^+$ T cells (Gate 2), $CD19^-$ $CD3^-$ $CD45^+$ non-B/non-T immune cells (Gate 3), and $CD19^-$ $CD3^-$ $CD45^-$ non-immune stromal and epithelial cells (Gate 4). Gating into T cells is also shown in Figure EV 2 and re-plotted here for convenience. **B)** UMAP embedding of annotated clusters in Gate 3.

Appendix Table S1: Cell culture

Antibody	Clone	Company	Final concentration
Purified anti-human CD3	UCHT1	BD Biosciences, Heidelberg, Germany	1 µg/mL
Purified anti-human CD28	CD28.2	BD Biosciences, Heidelberg, Germany	2 µg/mL
Ultra-LEAF^{TM} purified anti-human IgG1 isotype control	QA16A12	Biolegend, San Diego, USA	10 µg/mL
Ultra-LEAF^{TM} purified anti-human NKp80 (KLRF1)	5D12	Biolegend, San Diego, USA	10 µg/mL

Appendix Table S2: Flow stain KLRF1-receptor inhibition

Antigen	Fluorochrome	Clone	Company	Dilution
Extracellular staining				
CD8a	PerCP	RPA-T8	Biolegend, San Diego, USA	1/100
KLRB1 (CD161)	PE	191B8	Miltenyi Biotec, Bergisch Gladbach, Germany	1/50
KLRF1 (NKp80)	PE-Vio770	4A4.D10	Miltenyi Biotec, Bergisch Gladbach, Germany	1/50
KLRG1	APC	REA261	Miltenyi Biotec, Bergisch Gladbach, Germany	1/50
Intracellular staining				
CD3	BV785	OKT3	Biolegend, San Diego, USA	1/100
GPR56	VioBright FITC	REA467	Miltenyi Biotec, Bergisch Gladbach, Germany	1/10
IFN-γ	PE-Dazzle594	4S.B3	Biolegend, San Diego, USA	1/100
TNF	AF700	Mab11	Biolegend, San Diego, USA	1/200

Appendix Table S3: Flow stain AICL upregulation

.

Antigen	Fluorochrome	Clone	Company	Dilution
Extracellular staining				
*AICL (CLEC2B)**	PE		LSBio, Seattle, USA	1/20
CD8a	PerCP	RPA-T8	Biolegend, San Diego, USA	1/100
CD14	APC-A750	RMO52	Beckman Coulter, Brea, USA	1/50
CD56	BV605	HCD56	Biolegend, San Diego, USA	1/10
KLRB1 (CD161)	BV421	HP-3G10	Biolegend, San Diego, USA	1/20
KLRF1 (NKp80)	PE-Vio770	4A4.D10	Miltenyi Biotec, Bergisch Gladbach, Germany	1/50
KLRG1	APC	REA261	Miltenyi Biotec, Bergisch Gladbach, Germany	1/50
Intracellular staining				
*AICL (CLEC2B)**	PE		LSBio, Seattle, USA	1/20
CD3	BV785	OKT3	Biolegend, San Diego, USA	1/100
GPR56	VioBright FITC	REA467	Miltenyi Biotec, Bergisch Gladbach, Germany	1/10
IFN-γ	BV510	4S.B3	Biolegend, San Diego, USA	1/50
TNF	AF700	Mab11	Biolegend, San Diego, USA	1/200

Appendix Table S4: Sorting stain

.

Antigen	Fluorochrome	Clone	Company	Dilution
Extracellular staining				
*AICL (CLEC2B)**	PE		LSBio, Seattle, USA	1/50
CD8a	PerCP	RPA-T8	Biolegend, San Diego, USA	1/200
KLRF1 (NKp80)	PE-Vio770	4A4.D10	Miltenyi Biotec, Bergisch Gladbach, Germany	1/100
KLRG1	APC	REA261	Miltenyi Biotec, Bergisch Gladbach, Germany	1/50
CD3	BV785	OKT3	Biolegend, San Diego, USA	1/200
DAPI			Roche, Penzberg, Germany	1/250

Appendix Table S5: IMC panel

Table S4 IMC panel

Antigen	Isotope	Origin species	Company	Catalogue	Concentration (mg/mL)	Dilution
CD38	141Pr	Rabbit	Abcam	ab226034	0.2	1:500
CD19	142Nd	Rat	Invitrogen	14-0194-82	0.1	1:100
Granzyme B	143Nd	Rabbit	Abcam	ab219803	0.3	1:500
KLRF1	144Nd	Rabbit	Abcam	ab198928	0.1	1:100
β-Actin	145Nd	Mouse	Santa Cruz	sc-47778	0.4	1:400
HLA-DR	146Nd	Mouse	Invitrogen	14-9956-82	0.3	1:500
GPR56	147Sm	Mouse	Santa Cruz	sc-390192	0.15	1:100
CD107a	148Nd	Rabbit	Cell Signalling	9091BF	0.15	1:500
p53	149Sm	Mouse	Cell Signalling	48818	0.4	1:100
PD-L1	150Nd	Rabbit	Fluidigm	3150031D	0.5	1:200
CD31	151Eu	Rabbit	Fluidigm	3151025D	0.5	1:50
CD45	152Sm	Rabbit	Fluidigm	3152018D	0.5	1:100
CD103	153Eu	Rabbit	Abcam	ab271889	0.3	1:100
Tim-3	154Sm	Rabbit	Fluidigm	3154024D	0.5	1:200
KLRG1	155Gd	Rabbit	R&D Systems	MAB70293	0.2	1:100
CD4	156Gd	Rabbit	Abcam	ab181724	0.3	1:500
CD324	158Gd	Rabbit	Fluidigm	3158029D	0.5	1:400
CD68	159Tb	Mouse	Invitrogen	17-0688-82	0.3	1:200
T-Bet	160Gd	Rabbit	Cell Signalling	13232	0.2	1:500
CA19-9	161Dy	Mouse	Abcam	ab3982	0.1	1:300

CD8	162Dy	Mouse	Biolegend	372902	0.3	1:50
AICL	163Dy	Rabbit	Abcam	ab235626	0.3	1:200
Ki67	164Dy	Mouse	BD Biosciences	556003	0.05	1:100
PD-1	165Ho	Rabbit	Abcam	186928	0.5	1:100
CTLA-4	166Er	Rabbit	Abcam	ab237712	0.1	1:100
CD11c	167Er	Rabbit	Abcam	ab216655	0.4	1:500
CD127	168Er	Rabbit	Fluidigm	3168026D	0.5	1:100
TCR delta	169Tm	Mouse	Santa Cruz	sc-100289	0.2	1:50
CD3	170Er	Rabbit	Cell Signalling	85061BF	0.2	1:500
CD69	171Yb	Rabbit	Abcam	ab234512	0.2	1:500
CK18	172Yb	Mouse	Abcam	ab7797	0.4	1:200
FoxP3	173Yb	Mouse	Invitrogen	14-4777-82	0.3	1:100
aSMA	174Yb	Mouse	Abcam	ab240654	0.2	1:2000
CD25	175Lu	Rabbit	Fluidigm	3175036D	0.5	1:100
Histone H3	176Yb	Rabbit	Fluidigm	3176023D	0.5	1:600
DNA Intercalator	191Ir 193Ir	-	Fluidigm	201192A	0.2	1:400

(cont. App. Table S5)

Appendix Table S6: IMC Metadata

ROI	Ablation_ID	ROI_ID	Tissue	Batch	Patient_ID	TumorType
IM_20220623_OT3_001	IM_20220623_OT3_s0_p4_r1_a1_ac	1	Lung	OT3	P018	Tumor
IM_20220623_OT3_002	IM_20220623_OT3_s0_p4_r2_a2_ac	2	Lung	OT3	P018	Healthy
IM_20220623_OT3_003	IM_20220623_OT3_s0_p4_r3_a3_ac	3	Lung	OT3	P019	Tumor
IM_20220623_OT3_004	IM_20220623_OT3_s0_p4_r4_a4_ac	4	Lung	OT3	P019	Healthy
IM_20220623_OT3_005	IM_20220623_OT3_s0_p4_r5_a5_ac	5	Lung	OT3	P023	Tumor
IM_20220623_OT3_006	IM_20220623_OT3_s0_p4_r6_a6_ac	6	Lung	OT3	P023	Healthy
IM_20220623_OT3_007	IM_20220623_OT3_s0_p4_r7_a7_ac	7	Lung	OT3	P024	Tumor
IM_20220623_OT3_008	IM_20220623_OT3_s0_p4_r8_a8_ac	8	Lung	OT3	P024	Healthy
IM_20220623_OT3_009	IM_20220623_OT3_s0_p4_r9_a9_ac	9	Lung	OT3	P027	Tumor
IM_20220623_OT3_010	IM_20220623_OT3_s0_p4_r10_a10_ac	10	Lung	OT3	P027	Healthy
IM_20220623_OT3_011	IM_20220623_OT3_s0_p4_r11_a11_ac	11	Tonsil	OT3	NA	NA

Appendix Table S7: scRNA-seq IFNg primer

seq_id	seq_len	start	end	primer_len	primer_id	penalty	sequence
IFNG	1084	887	907	21	IFNG.primer_left_4	2,544528	ACTAGGCAGCCAACCTAAGCA

tm	gc_percent	self_any_th	self_end_th	hairpin_th	end_stability	pcr_product_size	intergenic_off_targets	intronic_off_targets	exonic_off_targets
61,455	52,381	15,8	0	39,29	3,91	278	0	0	0

Appendix Table S8: Gene sets used to calculate Tpex and Tex scores in Figure EV 5

TpexScore	TCF7	TexScore	TOX
	CXCR5		GZMB
	TBX21		HAVCR2
	WNT2		CTLA4
	MKI67		TIGIT
	IFNG		LAG3
	TNF		EOMES
	SELL		PRDM1
	BCL2		PDCD1
	CCR7		CX3CR1
	MYB		GZMH
	CD28		GNLY
	CD27		NFATC1
	BCL6		IRF4
	FAS		MTOR
	FOXO1		BATF
	TNFRSF9		NR4A1
	LEF1		NR4A2
	BACH2		NR4A3
	GNG4		ENTPD1
	CD200		CD244
	IL7R		CD38
	SATB1		PRF1
	IL6ST		
	GZMK		
	SLAMF6		
	ID3		
