# Supplementary material for: The AICL-KLRF1 axis supports CD4-CD8 T cell communication and cytokine competence in pre-exhausted CD8+ T cells
Source: EMBO Rep. 2026 Mar 18;27(8):2029–60. doi: 10.1038/s44319-026-00732-5 (PMC13121605; doi:10.1038/s44319-026-00732-5)
Supplement: Supplementary file 6 — Expanded View Figures [file 44319_2026_732_MOESM6_ESM.pdf]

Expanded View Figures

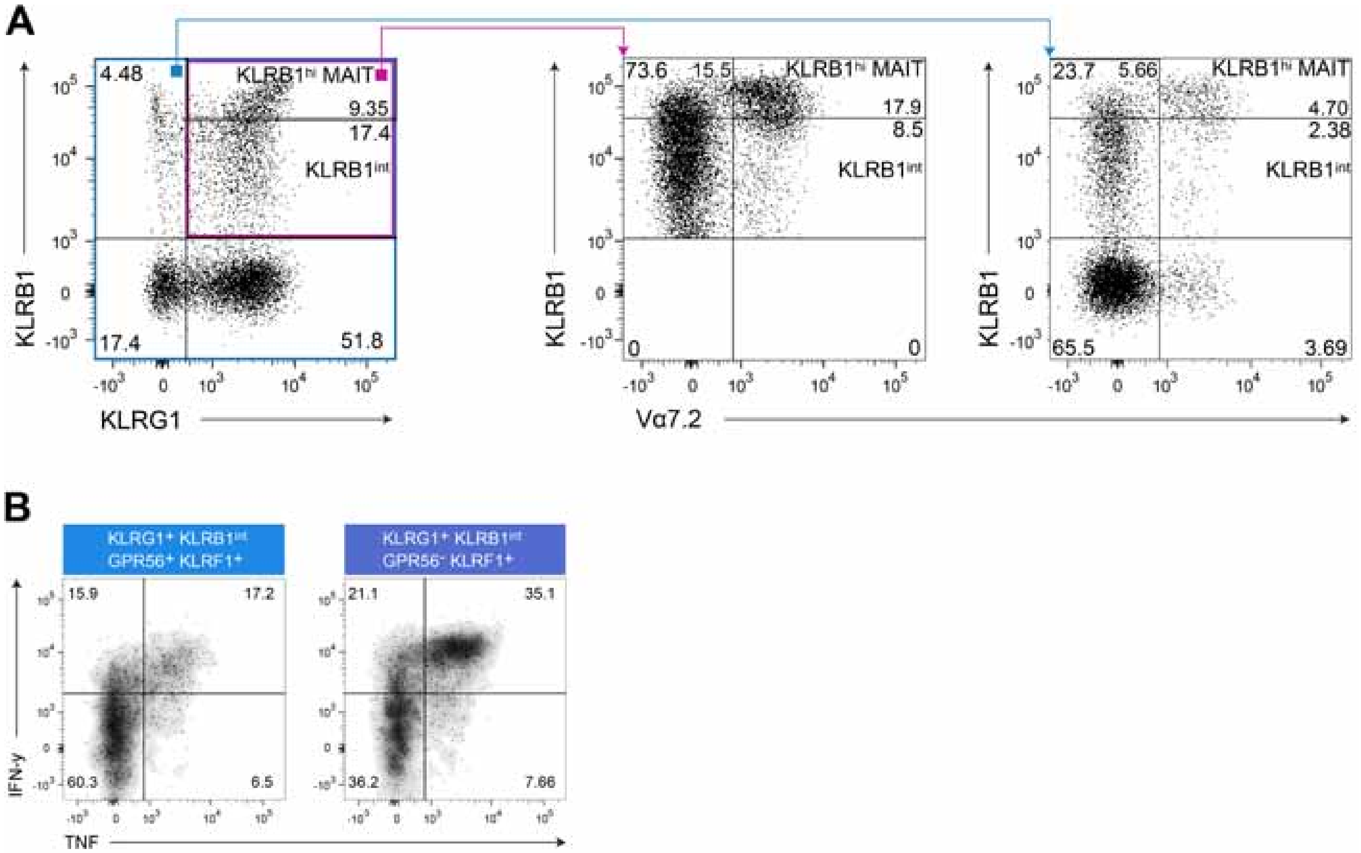

Figure EV1. Identification and exclusion of KLRBhi CD8^{+} MAIT cells from the analysis in Fig. 1.

(**A**) Exemplary dot plots showing KLRB1 versus Vα7.2 staining of pre-gated KLRB1^{+} cells of CD8^{+} T cells from PBMC to identify and exclude KLRB1^{high}-expressing MAIT cells. (**B**) Exemplary dot plots belonging to the analysis in Fig. 1D showing the TNF and IFN-γ producing CD8^{+} KLRG1^{+} KLRB1^{int} GPR56^{+} KLRF1^{+} T cells compared to their GPR56^{-} counterparts. An additional shading was added to the dot plots to highlight the populations.

 © The Author(s)

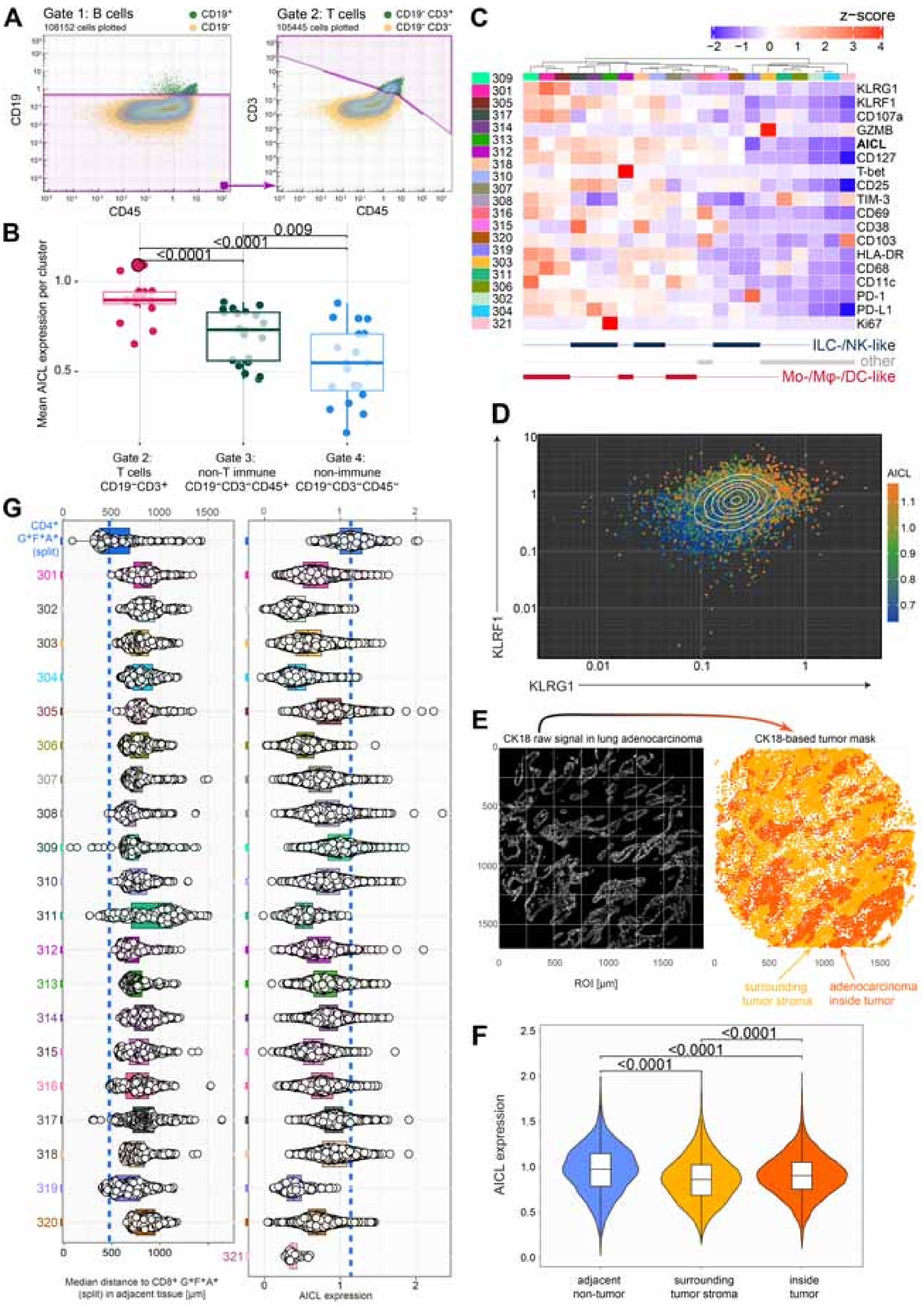
A
Gate 1: B cells
CD19+
CD19-
108152 cells plotted
CD19
CD45
Gate 2: T cells
CD19- CD3+
CD19- CD3-
105445 cells plotted
CD3
CD45
B
Mean AICL expression per cluster
<0.0001
<0.0001
0.009
Gate 2: T cells CD19−CD3+
Gate 3: non-T immune CD19−CD3−CD45+
Gate 4: non-immune CD19−CD3−CD45−
C
z−score
KLRG1
KLRF1
CD107a
GZMB
AICL
CD127
T-bet
CD25
TIM-3
CD69
CD38
CD103
HLA-DR
CD68
CD11c
PD-1
PD-L1
Ki67
ILC-/NK-like
other
Mo-/Mφ-/DC-like
D
AICL
KLRF1
KLRG1
E
CK18 raw signal in lung adenocarcinoma
CK18-based tumor mask
ROI [µm]
surrounding tumor stroma
adenocarcinoma inside tumor
F
AICL expression
<0.0001
<0.0001
<0.0001
adjacent non-tumor
surrounding tumor stroma
inside tumor
G
CD4+ G+F+A+ (split)
Median distance to CD8+ G+F+A+ (split) in adjacent tissue [µm]
AICL expression

Figure EV2. IMC cell gating strategy, quality control, and analysis of the non-B/non-T immune cell subset.

(**A**) Boolean gating strategy employed to identify T cells ($CD19^-$ $CD3^+$) used for this study. B cells were removed by gating on CD19 marker expression, and the remaining cells were gated for $CD3^+$ $CD45^+$ double-positive cells using the polygonal line as shown. (**B**) AICL expression across the T cell (Gate 2), non-B/non-T immune cell (Gate 3), and remaining non-immune stromal and epithelial cell (Gate 4) clusters in tumor and adjacent tissue samples combined. Plotted are cluster-wise mean AICL expressions of all 16 (Gate 2), 20 (Gate 3), and 19 (Gate 4) clusters ($n = 5$, biological replicates). In Gate 2, the $CD4^+CD8^+$ $G^+F^+A^+$ T cell cluster is highlighted with a bigger dot size. Statistical analysis was performed by the Wilcoxon rank-sum test with Benjamini and Hochberg adjustment. Exact *p* values are noted in the plot. Box plots show the median (center line) and interquartile range (box limits 25th to 75th percentile, 1x IQR), whiskers extend to the farthest values but no more than 1.5x IQR from the box. (**C**) Heatmap displaying *z*-scored marker expression of the identified non-B/non-T immune cell clusters (Gate 3). Cluster assignment is shown on the bottom of the heatmap: (blue) ILC-/NK-like: innate lymphoid and natural killer cell-like; (red) myeloid clusters Mo-/Mφ-/DC-like: monocyte-, macrophage-, dendritic cell-like; (gray) other: other immune cell clusters. (**D**) Dot plot of KLRG1 versus KLRF1 marker expressions of all gated $CD3^+$ T cells, color-coded by AICL marker expression (color gradient clipped at 15th to 85th percentile of AICL expression). (**E**) The CK18 raw signal was used to create lung adenocarcinoma tumor masks for each ROI. The masks were loaded into R to map cell centroid positions. Shown is a representative ROI with the CK18 signal (white) aligned with the cell positions marked in red/orange for cells mapped inside/outside of the binary mask. The same color-code was also used in Fig. 5D. (**F**) AICL expression of $CD4^+$ T cells in adjacent non-tumor samples (blue). Cells in lung adenocarcinoma tissue are sectioned into tumor (red) and surrounding tumor stroma (orange). Same color-code as in panel (**E**); $n = 5$, biological replicates. Statistical analysis was performed with a two-tailed *t*-test with Benjamini and Hochberg adjustment. Exact *p* values are noted in the plot. Box plots show the median (center line) and interquartile range (box limits 25th to 75th percentile, 1x IQR), whiskers extend to 1.5x IQR from the box **G**) Left: Neighborhood analysis of cells belonging to the split $CD8^+$ $G^+F^+A^+$ cluster probing median distances to $CD4^+$ $G^+F^+A^+$ T cells (split, see Fig. 5A) and cells of the non-B/non-T immune cell clusters (Gate 3, panel **C**). Right: AICL expression of the cells probed in the neighborhood analysis. Box plots show the median (center line) and interquartile range (box limits 25th to 75th percentile, 1x IQR), whiskers extend to 1.5x IQR from the box. Median distances and AICL expression of the split $CD4^+$ $G^+F^+A^+$ T cells are highlighted as a blue dashed line in the corresponding plots (both plots $n = 5$, biological replicates).

 © The Author(s)

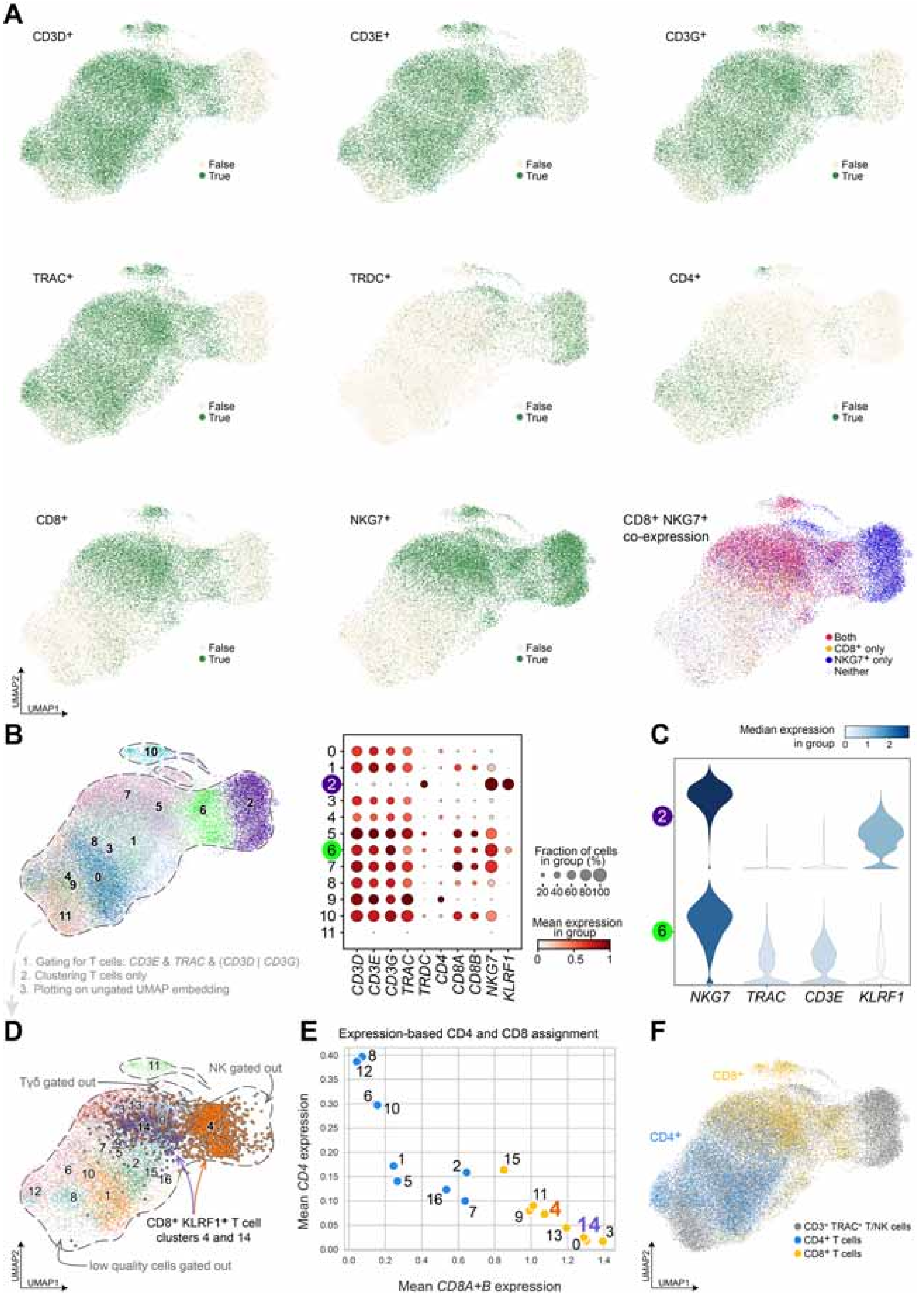
A
CD3D+
CD3E+
CD3G+
TRAC+
TRDC+
CD4+
CD8+
NKG7+
CD8+ NKG7+ co-expression
False
True
Both
CD8+ only
NKG7+ only
Neither
UMAP2
UMAP1
B
C
Median expression in group
Fraction of cells in group (%)
Mean expression in group
1. Gating for T cells: CD3E & TRAC & (CD3D | CD3G)
2. Clustering T cells only
3. Plotting on ungated UMAP embedding
CD3D CD3E CD3G TRAC TRDC CD4 CD8A CD8B NKG7 KLRF1
NKG7 TRAC CD3E KLRF1
D
Tγδ gated out
NK gated out
CD8+ KLRF1+ T cell clusters 4 and 14
low quality cells gated out
E
Expression-based CD4 and CD8 assignment
Mean CD4 expression
Mean CD8A+B expression
F
CD8+
CD4+
CD3+ TRAC+ T/NK cells
CD4+ T cells
CD8+ T cells

Figure EV3. scRNA-seq T cell gating strategy of annotated NK/T cells in Bischoff et al.

(**A**) Markers typically associated with NK cells (*NKG7*), γδ T cells (*TRDC*), and T cells (*CD3*, *CD4*, *CD8*, and *TRAC*) were mapped onto the UMAP embedding, with each marker plotted separately. Cells expressing these genes are highlighted in green. Bottom right: Cells expressing both *CD8* and *NKG7* are shown in red, while cells with single reads of either *CD8* (yellow) or *NKG7* (blue) are distinguished. (**B**) Clustering of the entire NK/T cell space predominantly yielded a T cell cluster (6, green) and an NK cell cluster (2, violet). A dot plot highlights a notable proportion of $TRDC^{+}$ and $NKG7^{+}$ impurities within cluster 6. (**C**) Violin plots depict gene expression of NK and T cell markers in clusters 2 and 6 of ungated NK/T cells, showing that cluster 6, primarily composed of T cells, expresses lower KLRF1 levels than cluster 2. (**D**) The NK/T cell subset was gated on T cells expressing $CD3E^{+}$ and $TRAC^{+}$ along with either $CD3D^{+}$ or $CD3G^{+}$. The gated T cells were re-clustered and plotted using the UMAP coordinates from panel B. The $KLRF1^{+}$ clusters 14 and 4, referenced in Fig. 6, are highlighted. (**E**) T cell clusters were assigned based on *CD4* and *CD8* expression and color-coded by main lineage, with clusters 14 and 4 ($KLRF1^{+}$) highlighted as in Fig. 6. (**F**) Using the color scheme from panel (**E**), $CD4^{+}$ (blue) and $CD8^{+}$ (yellow) T cells are plotted along with gated-out cells (gray).

 © The Author(s)

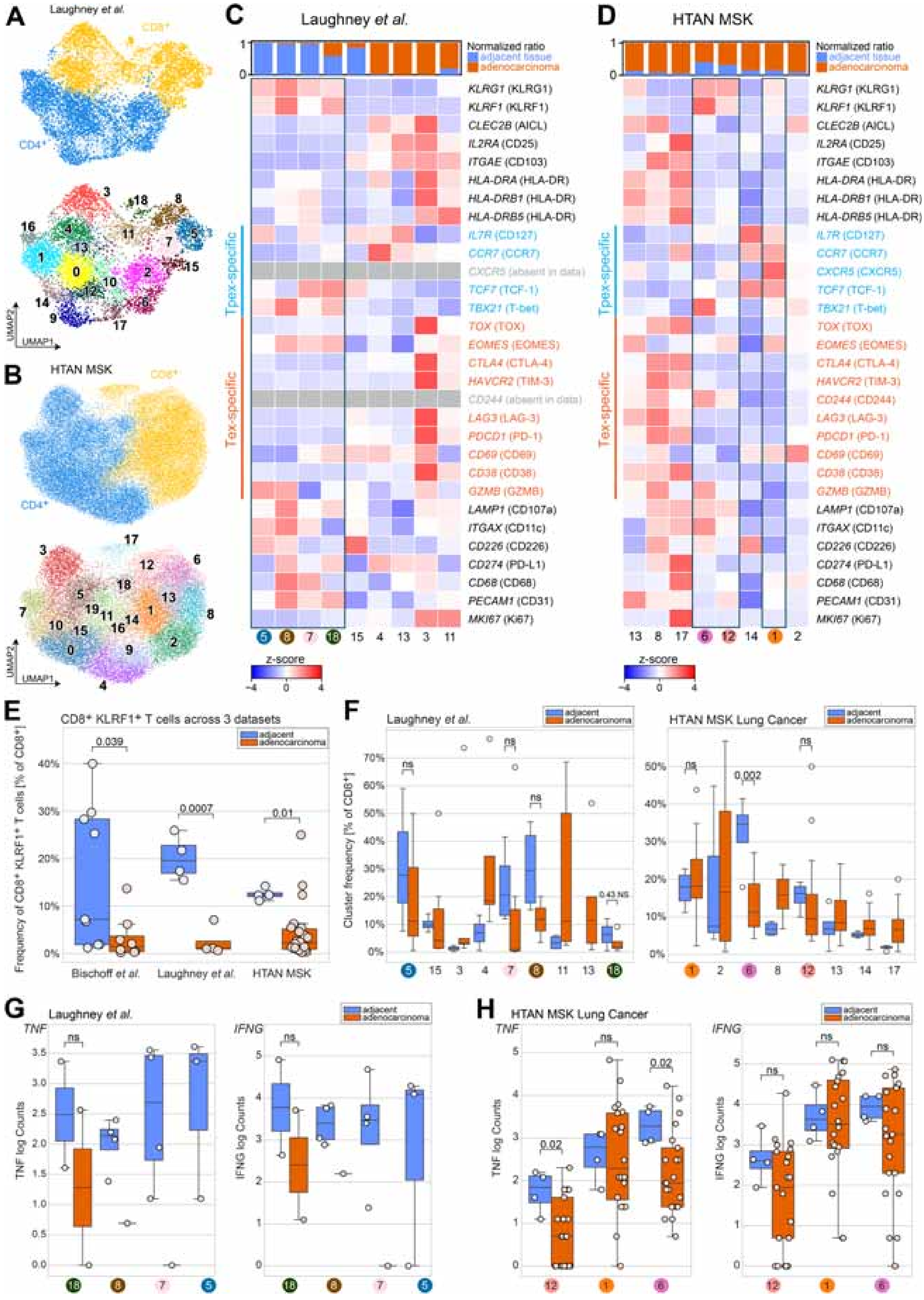
A
Laughney et al.
B
HTAN MSK
C
Laughney et al.
D
HTAN MSK
Tpex-specific
Tex-specific
E
CD8+ KLRF1+ T cells across 3 datasets
F
Laughney et al.
HTAN MSK Lung Cancer
G
Laughney et al.
H
HTAN MSK Lung Cancer

Figure EV4. CD8$^+$ T cell analysis in Laughney et al and HTAN MSK lung cancer scRNA-seq datasets.

Both datasets (Laughney et al, 2020; Chan et al, 2021) were gated using the same strategy used throughout the manuscript by identifying T cells based on *CD3E*>0 and (*CD3D*>0 | *CD3G*>0) together with *TRAC*>0. (Figs. 6 and EV3B,D) to exclude NK cells before analysis. (**A**, **B**) UMAP embeddings of both datasets, color-coded by major CD4$^+$ and CD8$^+$ lineage markers and Leiden clusters. (**C**, **D**) Heatmaps of CD8$^+$ T cell clusters identified in both datasets, with markers grouped by activation and exhaustion. KLRF1$^+$ clusters are highlighted with boxes. (**E**) Frequencies of *KLRF1*$^+$ CD8$^+$ T cells relative to all CD8$^+$ T cells in all three datasets, separated into carcinoma tissue (orange) and adjacent non-tumor (blue) tissue samples (Bischoff $n = 12$, Laughney $n = 9$, HTAN $n = 26$, all biological replicates). Statistical analysis was performed by the Wilcoxon rank-sum test. Exact p values are noted in the plot. Box plots show the median (center line) and interquartile range (box limits 25th to 75th percentile, 1x IQR), whiskers extend to the farthest values but no more than 1.5x IQR from the box. All data points are drawn. (**F**) Patient-wise frequencies of CD8$^+$ T cell clusters relative to all CD8$^+$ T cells, separated by carcinoma (orange) and adjacent non-tumor normal (blue) tissue (Laughney $n = 9$, HTAN $n = 26$, both datasets using biological replicates). Statistical analysis was performed with a one-tailed Wilcoxon rank-sum test. Exact p value is noted in the plot; ns $p > 0.05$. Box plots show the median (center line) and interquartile range (box limits 25th to 75th percentile, 1x IQR), whiskers extend to the farthest values but no more than 1.5x IQR from the box. Only data points beyond the whiskers are drawn. (**G**, **H**) Cytokine production of CD8$^+$ KLRF1$^+$ T cell clusters, separated by carcinoma (orange) and adjacent non-tumor normal (blue) tissue (Laughney $n = 9$, HTAN $n = 26$, both biological replicates). Statistical analysis was performed by a one-tailed Wilcoxon rank-sum test. Exact p values are noted in the plot; ns $p > 0.05$. Box plots show the median (center line) and interquartile range (box limits 25th to 75th percentile, 1x IQR), whiskers extend to the farthest values but no more than 1.5x IQR from the box. All data points are drawn.

 © The Author(s)

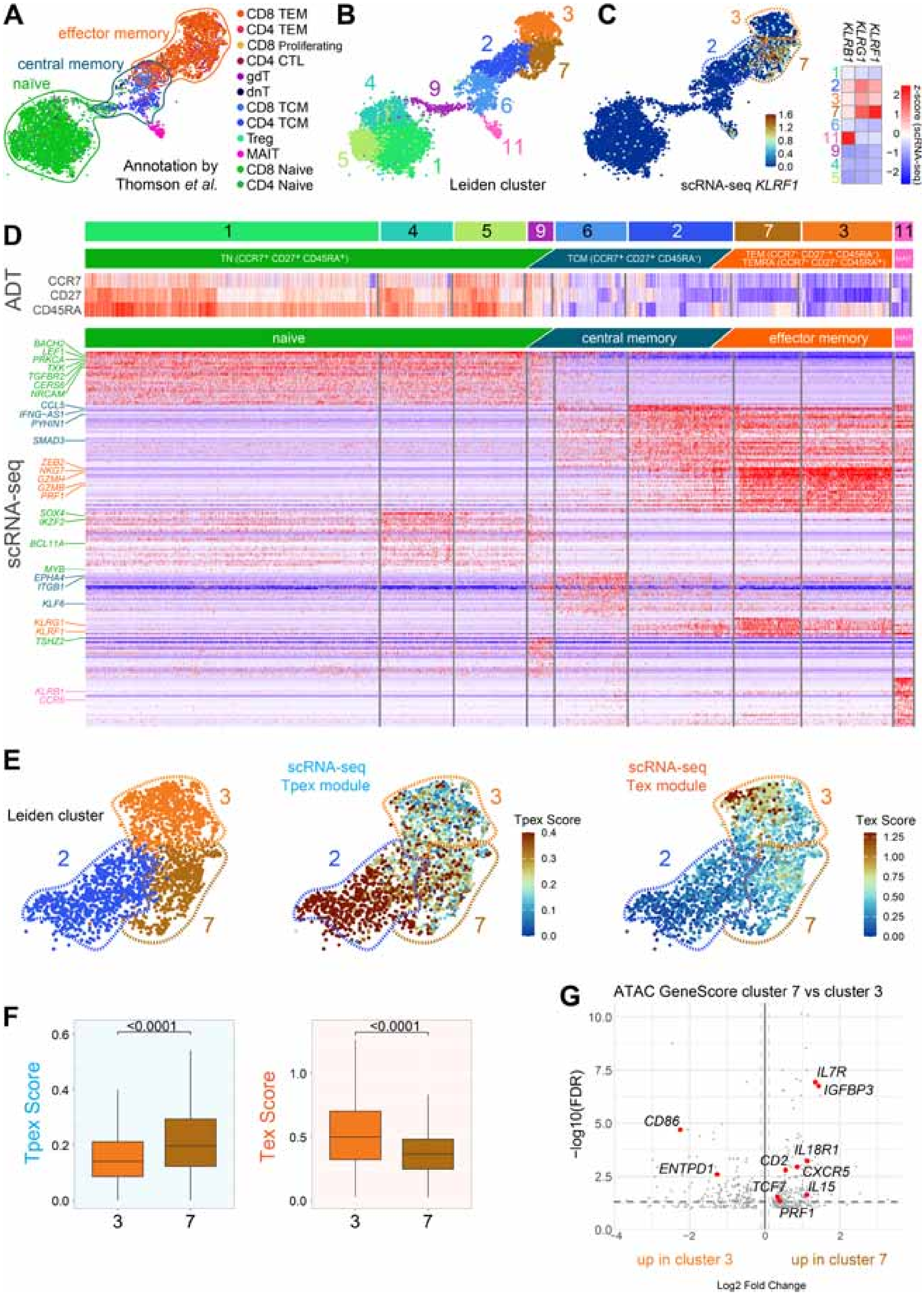
A
effector memory
central memory
naive
Annotation by Thomson et al.
CD8 TEM
CD4 TEM
CD8 Proliferating
CD4 CTL
gdT
dnT
CD8 TCM
CD4 TCM
Treg
MAIT
CD8 Naive
CD4 Naive
B
Leiden cluster
C
scRNA-seq KLRF1
KLRF1
KLRG1
KLRB1
z-score (scRNA-seq)
D
ADT
CCR7
CD27
CD45RA
naive
central memory
effector memory
scRNA-seq
BACH2
LEF1
PRKCA
TXK
TGFBR2
CERS6
NRCAM
CCL5
IFNG-AS1
PYHIN1
SMAD3
ZEB2
NKG7
GZMH
GZMB
PRF1
SOX4
IKZF2
BCL11A
MYB
EPHA4
ITGB1
KLF6
KLRG1
KLRF1
TSHZ2
KLRB1
CCR6
E
Leiden cluster
scRNA-seq Tpex module
Tpex Score
scRNA-seq Tex module
Tex Score
F
Tpex Score
<0.0001
Tex Score
<0.0001
G
ATAC GeneScore cluster 7 vs cluster 3
-log10(FDR)
IL7R
IGFBP3
CD86
IL18R1
ENTPD1
CD2
CXCR5
TCF7
IL15
PRF1
up in cluster 3
up in cluster 7
Log2 Fold Change

Figure EV5. CD8$^+$ T cell analysis in Thomson et al trimodal scRNA-seq, ADT, and ATAC dataset.

The dataset (Thomson et al, 2023) was gated using an extended version of the gating strategy to identify CD8$^+$ T cells while excluding CD4$^+$ and γδ T cells (see methods part). (**A**) UMAP embedding of gated CD8$^+$ T cells, color-coded by the predicted T cell types as reported by Thomson et al (predicted.t-celltype.l2). Differentiation stages are colored in similar tones: naïve (green), central memory T cells (TCM, blue), mucosal-associated invariant T cells (MAIT, pink), effector memory (TEM) and cytotoxic T lymphocytes (CTL, both orange). Minor populations were colored separately: regulatory T cells (Treg, aquamarine), double-negative T cells (dnT, black), γδ T cells (gdT, turquoise). (**B**) UMAP embedding color-coded by Leiden clusters determined by the weighted-nearest neighbor (WNN) graph constructed from scRNA-seq PCA and ATAC LSI embeddings. (**C**) UMAP embedding color-coded by the KLRF1 expression and a z-scored heatmap of KLRs transcription levels used in our study. (**D**) Antibody-derived tags used in Thomson et al to gate into naïve T cell subsets plotted as a z-scored heatmap across the calculated Leiden clusters. Bottom: Differential gene expression list with top 40 genes per cluster. Genes used to align and annotate the clusters are highlighted in the same color-code as in panel (**A**). (**E**) UMAP embeddings of Tex and Tpex module scores calculated from the gene sets shown in the Appendix Table S8 (**F**) Comparison of Tpex and Tex scores of the clusters 3 and 7 ($n = 16$, biological replicates. Cluster 3 730 cells; cluster 7 530 cells). Statistical analysis was performed by a two-tailed *t*-test. Exact *p* values are noted in the plot. Box plots show the median (center line) and interquartile range (box limits 25th to 75th percentile, 1x IQR), whiskers extend to the farthest values but no more than 1.5x IQR from the box. (**G**) Differential ATAC gene scores between clusters 7 and 3. Gene scores enriched in the KLRF1$^+$ cluster 7 are log2FC >0. Loci associated with memory/Tpex states and exhaustion, and FDR <0.05 are highlighted.

 © The Author(s)
